# Growth Factor Release within Liquid and Solid PRF

**DOI:** 10.3390/jcm11175070

**Published:** 2022-08-29

**Authors:** Katharina Zwittnig, Barbara Kirnbauer, Norbert Jakse, Peter Schlenke, Irene Mischak, Shahram Ghanaati, Sarah Al-Maawi, Dániel Végh, Michael Payer, Tomislav A. Zrnc

**Affiliations:** 1 Division of Oral Surgery and Orthodontics, Department of Dental and Oral Health, Medical University of Graz, Billrothgasse 4, 8010 Graz, Austria; 2 Department of Blood Group Serology and Transfusion Medicine, Medical University of Graz, 8036 Graz, Austria; 3 Department for Oral, Cranio-Maxillofacial and Facial Plastic Surgery, FORM (Frankfurt Orofacial Regenerative Medicine) Lab, University Hospital Frankfurt, Goethe University, Theodor-Stern-Kai 7, 60590 Frankfurt am Main, Germany; 4 Department of Prosthodontics, Semmelweis University, 1088 Budapest, Hungary; 5 Division of Oral and Maxillofacial Surgery, Department of Dental Medicine and Oral Health, Medical University of Graz, Billrothgasse 4, 8010 Graz, Austria

**Keywords:** oral surgery, platelet-rich fibrin, growth factors, VEGF, TGF, MMP-9, EGF, PDGF-BB

## Abstract

*Aim:* The purpose of this study was to obtain data concerning growth factor release within liquid and solid platelet-rich fibrin (PRF) matrices and to estimate the amount of potential interindividual variations as a basis for further preclinical and clinical trials. Therefore, we aimed to determine possible differences in the release of growth factors between liquid and solid PRF. *Materials and Methods:* Blood samples obtained from four subjects were processed to both liquid and solid PRF matrices using a standard centrifugation protocol. Five growth factors (vascular endothelial growth factor, VEGF; epidermal growth factor, EGF; platelet-derived growth factor-BB, PDGF-BB; transforming growth factor-β1, TGF-β1; and matrix metallopeptidase 9, MMP-9) have been evaluated at six time points by ELISA over a total observation period of 10 days (1 h, 7 h, 1 d, 2 d, 7 d, and 10 d). *Results:* Growth factor release could be measured in all samples at each time point. Comparing liquid and solid PRF matrices, no significant differences were detected (*p* > 0.05). The mean release of VEGF, TGFβ-1, PDGF-BB, and MMP-9 raised to a peak at time point five (day 7) in both liquid and solid PRF matrices. VEGF release was lower in liquid PRF than in solid PRF, whereas those of PDGF-BB and MMP-9 were higher in liquid PRF than in solid PRF at all time points. EGF had its peak release already at time point two after 7 h in liquid and solid matrices (hour 7 EGF solid: mean = 180 pg/mL, SD = 81; EGF liquid: mean = 218 pg/mL, SD = 64), declined rapidly until day 2, and had a second slight peak on day 7 in both groups (day 7 EGF solid: mean = 182 pg/mL, SD = 189; EGF liquid: mean = 81 pg/mL, SD = 70). *Conclusions:* This study detected growth factor release within liquid and solid PRF matrices with little variations. Further preclinical trials are needed to precisely analyze the growth factor release in larger samples and to better understand their effects on wound healing in different clinical indications.

## 1. Introduction

Blood concentrates have emerged as an integral technique in dentistry in the past couple of years. The purpose of using such autologous treatment is to support natural healing, accelerate tissue regeneration, and provide a more comfortable postoperative outcome for patients [1]. Platelet-rich plasma was invented in the 1980s [2,3]. Platelet-rich plasma is produced within two relatively time-consuming centrifugation processes with the addition of anticoagulants, such as bovine thrombin or Calcium chloride [1,4]. Disagreement on a protocol and many different kits marketed has hampered comparability over the years [4]. Anitua et al. then went on to innovate platelet-rich growth factors [5]. Platelet-rich fibrin (PRF) was developed in 2006 and first published by Choukroun et al. [6].

PRF is probably the most well-known blood-derived product because of its fast and cost-effective preparation method. With one-step centrifugation, it is possible to create ready-for-use PRF matrices [7,8,9]. However, since no anticoagulants are administered in PRF creation, blood samples must be centrifuged immediately after blood collection [1]. In order to ensure reproducibility, characteristics have been determined. This is intended to create transparency and clarity [10], and should facilitate the comparability of studies and, consequently, the path to evidence-based knowledge. PRF has been used in oral surgery for almost 20 years [11]. It has also drawn attention from other disciplines, such as plastic surgery. In general, it is occasionally applied for the treatment of chronic wounds and diabetic ulcers [12,13,14]. In order to support the natural healing process of soft tissue and avoid infections, it is applied in the management of burns [14].

The benefits of PRF regarding wound healing have become well known in the meantime, whereas the interest in this is still rising [15]. Clinical awareness of this additive-free blood-derived product has increased continuously. One decisive factor for the regenerative potential of PRF could be its capacity to release different growth factors. In addition to the activated platelets and leukocytes, the fibrin network acts as a reservoir for growth factors, enabling a continuous release profile [9,16,17,18,19]. Particularly, vascular endothelial growth factor (VEGF), platelet-derived growth factor-BB (PDGF), and transforming growth factor-β1 (TGFβ-1) are considered promoters of wound healing and can be detected in the fibrin network [20]. Generally, PRF offers various opportunities for clinical application in dentistry [21,22,23].

Individual clinical situations require different types of application [23,24]. Therefore, this blood-derived product can be individually adjusted and prepared according to specific clinical requirements as both liquid and solid matrices of PRF. Additionally, if required, solid matrices can be manufactured to create membranes [19] that may be used in guided bone regeneration approaches within dental implant therapy [25]. Furthermore, cylindric solid matrices can be created as clots to fill extraction sockets to prevent patients from suffering from severe alveolar ridge resorption and alveolus osteitis [26]. It has been shown that the pain level was low and soft tissue healing was promoted after extractions [27]. Liquid PRF can be used to biologize biomaterials, such as bone substitutes and xenogeneic collagen membranes [18]. The literature even reports on the injection of liquid PRF into the temporomandibular joint for the treatment of craniomandibular dysfunction [28]. Especially for patients with a risk of medication-related osteonecrosis of the jaw and, respectively, patients suffering from MRONJ, the platelet concentrate seems to provide a benefit. With regard to both the prevention of MRONJ in patients at risk and the addition of PRF to conventional therapies at all stages of MRONJ, positive reports are found in the literature. However, there is a lack of large studies to support the evidence [29,30,31]. The consistency of PRF may, however, influence the amount and kinetics of growth factor release. Growth factor release within PRF can be evaluated using ELISA assays [9,19,32,33]. Sandwich ELISA was used in this study. Growth factors are detected as they bind with the capture antibodies located on the 96-well plate, as well as with the detection antibodies added. Due to addition of a substrate, there is a reaction resulting in fluorescent activity. Thereby, the amount of specific growth factor can be detected [34]. In this context, the question arises as to whether both liquid and solid matrices release the same amount of growth factors over time, and whether there is a large variation between different individuals. To clarify this consideration also mentioned in previous studies [8,9,19,33], the present trial was initiated.

## 2. Materials and Methods

This pilot study was a result of cooperation between the Division of Oral Surgery and Orthodontics of the Department of Dental Medicine and Oral Health and the Department of Blood Group Serology and Transfusion Medicine, Medical University of Graz, Austria.

It was conducted according to the guidelines of the Declaration of Helsinki and approved by the Institutional Ethics Committee of the Medical University of Graz (protocol code: 31-495 ex 18/19 and approval date: 27 August 2019).

Four randomly selected, voluntary, healthy female subjects aged between 23 and 60 years participated in the study. As the trial was initiated as a pilot, free of funding, the study was limited to a number of 4 participants. In order to minimize the risk of bias due to different genders in this low number of participants, only female subjects were included in this first pilot study. A peripheral vein was punctured with a double wing cannula (Butterfly, APRF3042, Nice, France; Mectron, Cologne, Germany). Twenty milliliters of whole blood each was obtained, and 10 mL of whole blood was centrifuged in glass tubes (A-PRF tubes, Process for PRF™, Nice, France; Mectron, Cologne, Germany) and 10 mL in plastic tubes (i-PRF, Process for PRF™, Nice, France; Mectron, Cologne, Germany).

The centrifuge (Duo centrifuge, Process for PRF™, Nice, France; Mectron, Cologne, Germany) had a fixed angle, no brake, and a rotor size of 110 mm according to the protocol [16].

The centrifugation process lasted 8 min, with 1200 rpm and a relative centrifugal force (RCF) of 177 g [11,16]. The produced liquid and solid PRF were used to analyze the release of five growth factors for 10 days. As growth factor release tended to decrease at day 10 in previous studies, we decided to examine a period not longer than 10 days [19].

Two milliliters of liquid PRF was collected in a syringe and transferred to a six-well plate (Sarstedt^®^ cell culture plate 6-well) under sterile conditions. To force coagulation, it was incubated at 37 °C for 30 min. Meanwhile, solid PRF clots were separated bluntly from the erythrocytes below. Then, the PRF clots were placed onto a six-well plate (Sarstedt^®^ cell culture plate 6-well).

Medium (RPMI 1640+GlutaMax (500)) and antibiotic solution (streptomycin/ampicillin) were mixed. Five milliliters of the mixed solution was added to each solid PRF and each liquid PRF with a pipette (Eppendorf pipette Easypet^®^) under sterile conditions. A control sample, medium without PRF, was also set up.

Samples were incubated at 37 °C after six defined time points, and the supernatant was pipetted under sterile conditions. At every time point, the supernatant of each sample was split into six labeled, screwed tubes for further growth factor quantification (Sarstedt^®^ Micro-screw tube 2 mL, PP) to VEGF, PDGF-BB, epidermal growth factor (EGF), TGF-β1, matrix metallopeptidase 9 (MMP-9), and a control. Extracted liquid was replaced with 5 mL of fresh medium and then incubated at 37 °C. After 1 h, 7 h, 1 day, 2 days, 7 days, and 10 days, the process was repeated. Supernatants were frozen at −81 °C.

ELISA analysis was conducted according to the manufacturer’s manual using a sandwich ELISA (Duoset^®^ ELISA, R&D Systems). Samples were examined in triplicates. Statistical analysis was conducted using IBM SPSS Statistics Version 26 (IBM Corp., Armonk, NY, USA). A *p*-value of 0.05 was selected. Descriptive statistics will be applied to all outcomes. Independent Student *t*-tests were calculated to compare the means of the two groups.

## 3. Results

### 3.1. Growth Factor Analysis

**Figure 1:**
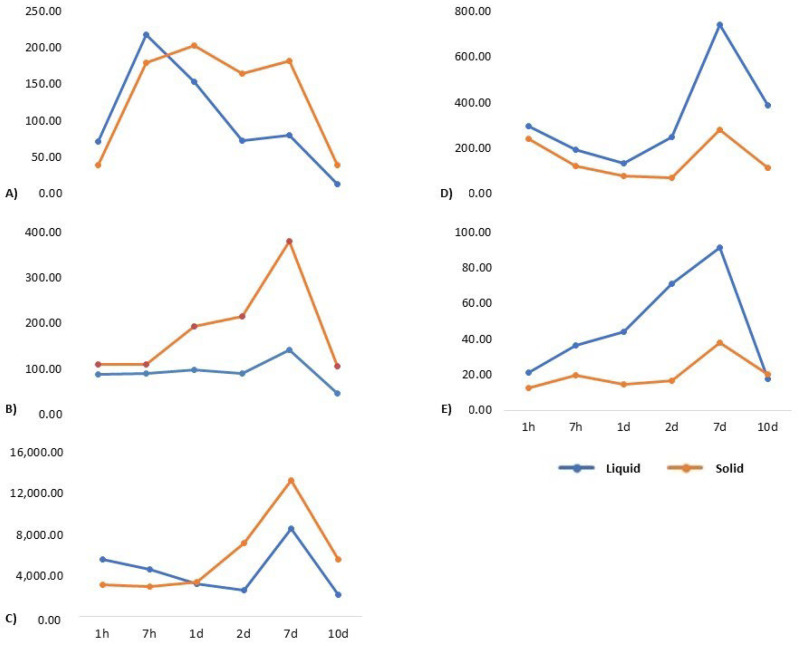
Growth factor release over time. Growth factor concentrations of liquid (blue) and solid (red) PRF over time for (**A**) EGF (pg/mL); (**B**) VEGF (pg/mL); (**C**) TGFβ-1 (pg/mL); (**D**) PDGF-BB (pg/mL); and (**E**) MMP-9 (ng/mL).

During the study, the release of five different growth factors of liquid and solid PRF was investigated at different time points (see Table 1 and Figure 1). The assessment of the growth factor release of PDGF, TGF-β1, VEGF, EGF, and MMP-9 in the supernatant after 1 and 7 h, as well as after 1, 2, 7, and 10 days, of incubation revealed the following:

#### 3.1.1. Liquid Matrices

- *Release of EGF*

**Figure 2:**
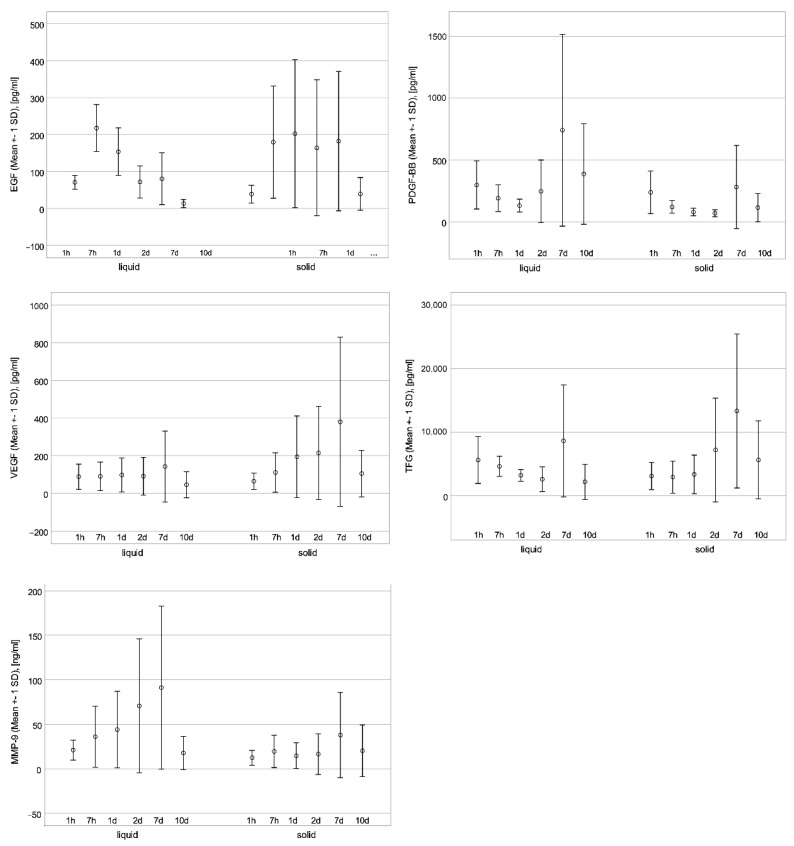
Growth factor release with error bars EGF (pg/mL), VEGF (pg/mL), TGFβ-1 (pg/mL), PDGF-BB (pg/mL), and MMP-9 (ng/mL).

After 1 h, an EGF concentration of 71 pg/mL in the supernatant was measured. The highest value of 218 pg/mL could be seen after 7 h. After a steep increase during the first 24 h, from day 2, EGF values decreased from 154 to 72 pg/mL and finally to 13 pg/mL on day 10 (Figure 2).

- *Release of VEGF*

VEGF release amounted constantly to approximately 90–100 pg/mL in the first 2 days. The highest release of VEGF in liquid PRF was observed on day 7 (142 pg/mL). Until day 10, the value decreased to 45 pg/mL (Figure 2).

- *Release of TGF-β1*

TGF-β1 started with 5619 pg/mL after 1 h and decreased constantly until day 2 to 2601 pg/mL. On day 7, an increase to 8639 pg/mL and, subsequently, a decrease to 2189 pg/mL on day 10 could be observed (Figure 2).

- *Release of PDGF-BB*

The first measurement of PDGF-BB after 1 h showed 297 pg/mL. It declined to 191 pg/mL already after 7 h and even to 131 pg/mL on day 1. On day 2, an increase to 247 pg/mL, similar to TGF-β1, was found. The highest value of PDGF-BB release could be observed on day 7 at 741 pg/mL. On day 10, 386 pg/mL could be measured (Figure 2).

- *Release of MMP-9*

MMP-9 analysis showed a constant increase from 21 ng/mL after 1 h to the highest value of 91 ng/pg on day 7. On day 10, only 17 ng/mL could be detected (Figure 2).

#### 3.1.2. Solid Matrices

- *Release of EGF*

Solid PRF released 39 pg/mL of EGF after 1 h, but increased to a plateau up to 203 pg/mL from 7 h to day 7. On day 10, it decreased to 40 pg/mL (Figure 2).

- *Release of VEGF*

The values of VEGF started at 110 pg/mL after 1 h and 110 pg/mL after 7 h. They increased to 194 pg/mL on day 1 and 215 pg/mL on day 2. Their maximum value was 380 pg/mL on day 7. On day 10, VEGF was 105 pg/mL (Figure 2).

- *Release of TGF-β1*

TGFβ-1 showed a release of 3111 pg/mL after 1 h, 2934 pg/mL after 7 h, and 3367 pg/mL after 24 h. It increased to 7211 pg/mL after 2 days and reached its peak on day 7 at 13,337 pg/mL. On day 10, a release of 5638 pg/mL could be observed (Figure 2).

- *Release of PDGF-BB*

The mean value of release for PDGF-BB was 238 pg/mL after 1 h and decreased steadily to its low point at 69 pg/mL on day 2. The highest level of PDGF-BB was measured on day 7 at 281 pg/mL. On day 10, it was 115 pg/mL (Figure 2).

- *Release of MMP-9*

MMP-9, again, was measured in nanogram per milliliter and had its maximum on day 7 at 38 ng/mL. The other time points were in a similar range between 12 ng/mL after 1 h and 20 ng/mL on day 10 (Figure 2).

### 3.2. Figures

### 3.3. Tables

**Table 1:** Descriptive statistics growth factor release of liquid and solid PRF.

			Consistency			
			Liquid		Solid	
Growth Factor	Time Point	N	Mean	SD	Mean	SD
EGF	1 h	4	71.1	18.7	38.9	23.8
[pg/mL]	7 h	4	217.9	63.7	179.7	152.0
	1 d	4	153.8	64.6	202.6	200.4
	2 d	4	72.0	43.4	164.2	184.0
	7 d	4	80.5	70.0	182.3	188.9
	10 d	4	13.3	11.2	39.6	44.3
MMP-9	1 h	4	21.1	11.2	12.5	8.3
[ng/pg]	7 h	4	36.1	34.3	19.6	18.1
	1 d	4	44.1	43.0	14.7	14.5
	2 d	4	70.7	75.2	16.5	22.9
	7 d	4	91.2	91.6	38.1	48.0
	10 d	4	17.7	18.7	20.3	29.1
PDGF-BB	1 h	4	296.9	193.9	238.1	172.0
[pg/mL]	7 h	4	191.1	108.3	119.6	51.2
	1 d	4	131.1	53.1	78.3	30.9
	2 d	4	246.6	253.3	69.3	28.4
	7 d	4	741.0	775.6	281.0	336.0
	10 d	4	386.5	406.2	114.5	114.4
TFG	1 h	4	5619.0	3686.2	3111.4	2154.6
[pg/mL]	7 h	4	4626.8	1571.1	2934.1	2531.2
	1 d	4	3220.4	911.3	3367.4	3023.7
	2 d	4	2601.1	1928.2	7211.2	8170.8
	7 d	4	8639.2	8795.0	13,337.4	12,093.7
	10 d	4	2189.0	2748.0	5638.2	6118.7
VEGF	1 h	4	88.1	67.1	64.0	43.5
[pg/mL]	7 h	4	89.9	75.8	110.4	105.0
	1 d	4	97.2	90.4	193.7	216.5
	2 d	4	90.3	99.4	214.6	246.9
	7 d	4	141.9	188.2	379.8	449.8
	10 d	4	45.4	69.6	104.7	123.8

## 4. Discussion

In summary, in this in vitro study, both liquid and solid matrices released growth factors for 10 days. The results highlighted the following findings: a peak on day 7 in all groups, as well as a decrease of all growth factor values until day 10. Only for EGF could the highest release be detected after 7 h in both solid and liquid matrices combined with a significant decrease between day 7 and day 10. These results are in accordance with other in vitro studies [8,19] and potentially reflected in clinical studies reporting favorable healing patterns and reductions of postoperative indisposition when using PRF in oral surgical interventions [23].

The namesake for PRF platelets has a versatile influence on wound healing. Additionally, platelets obtain a potential of immune cell activation, such as leukocytes and neutrophils, and attract them to the wound via pro-inflammatory and anti-inflammatory molecules [35]. Platelets’ anti-infective effects are based on the capacity of encapsulation of infectious agents and the formation of antimicrobial peptides [36]. Their α-granules contain VEGF, one of the investigated growth factors of the present trial, that supports vascular regeneration [37].

The bioactive capacity of PRF makes it of great interest in the context of surgical interventions throughout medical disciplines; however, its application has become popular also in oral surgery. Because of the availability of liquid and solid consistencies, PRF offers a wide variety of possible applications. Liquid matrices are used for the infiltration of, for example, the temporomandibular joint [38], whereas solid PRF is more likely to be used for socket preservations and to maintain the alveolar ridge after extractions [39]. Anwandter et al. reported the favorable recovery of extraction sockets when using PRF as a single filling material as a bone substitute. Additionally, the need for primary wound closure is smaller, as no particles of bone substitute are present and early exposition of collagen membranes related to wound dehiscence can be avoided [40].

Because of a lack of randomized controlled clinical trials using standardized protocols of PRF preparation, the evidence for significant effects of PRF in combination with other guided bone regeneration procedures, such as sinus floor augmentation, is, so far, not available. The bias caused by several factors, such as the application of different PRF preparation protocols and consistencies as well as different bone substitutes, complicates a clear statement regarding the effects of PRF in this context [26].

Another field of application resembles periodontology. Palatal donor sites of free gingival and connective tissue grafts provide great discomfort for patients. Patient-related outcome measures are significantly improved by the application of PRF as an alternative, as less pain and less bleeding are reported, and soft tissue healing appears to be equal or even improved [41].

In the present study, the growth factor release in liquid and solid PRF matrices has been examined and compared. Both matrices have been manufactured following the same protocol, according to Ghanaati et al. [16]. Referring to the presented results, the maximum release of PDGF-BB and MMP-9 is higher in liquid PRF samples (at day 7, 741 pg/mL versus 280 pg/mL for PDGF-BB, and 91 ng/mL versus 38 ng/mL for MMP-9). By contrast, the release of EGF, VEGF, and TGF-β1 tends to be higher in the solid PRF group. El Bagdadi et al. published an investigation of growth factor release in solid PRF matrices using a similar PRF protocol (1200 rpm for 8 min with an RCF of 177 g, a rotor size of 110 mm, and a rotor angulation of 41.3°) [8,16,42]. In their study, the highest concentration of TGF-β1 and VEGF was also released on day 7 and the highest amount of EGF in solid matrices was observed on day 1. These results were confirmed also in the present trial; however, the quantity of growth factor release in the study of El Bagdadi et al. differs in the concentration ranges. Concentrations of TGF-ß1 are higher at all time points, except for day 7, than those in the present investigation. [8]. These differences may be related to the low number of participants in both studies, which definitely has to be considered a drawback. Additionally, the number of platelets and the volume of the clot could influence the growth factor concentration.

Choukroun and Ghanaati measured, among other parameters, the release of TGF-β1 and VEGF after 1 h and 1 day using the exact same protocol (1200 rpm for 8 min with an RCF of 177 g, a rotor size of 110 mm, and a rotor angulation of 41.3°). The amount of TGF-β1 was higher than that of VEGF in their study in general. In our study, the same trend could be detected, but unfortunately, no measurements on days 7 and 10 were made to compare [9]. In our examination, day 7 was the time point with the highest amount of VEGF, TGF-β1, PDGF-BB, and MMP-9. The peak at time point five (day 7), however, presents an observation of utmost clinical relevance, as it demonstrates that high growth factor release is seen even several days after PRF application.

Another important finding of the study is that similar quantities of growth factors seem to be present in liquid and solid PRF consistencies.

The steady release of growth factors shown in the study suggests clinical significance in promoting the first days of wound healing when PRF is applied in both liquid and solid form.

Besides the low number of participants, another limitation of the present study is the fact that only female donors could be recruited. Therefore, and because of the large scatter, statistical analysis is thus limited to descriptive analysis.

Further studies with larger numbers of cases are needed to support these results.

## 5. Conclusions

There is no question that a study with this small number of cases allows only limited conclusions to be drawn. The study demonstrates that both liquid and solid PRF matrices release growth factors for 10 days. Interestingly, the release significantly decreased after day 7 in all examined growth factors in both groups and might contribute to biological stability for no longer than 7 days. The course between both groups is similar over the observation period. Based on the results of these investigations, it may be assumed that the capacity of growth factor release can be expected with solid and liquid PRF consistencies and that the decision of which PRF should be used can be based upon clinical requirements. Results, and therefore also the validity of the conclusion, are, however, limited because of the small sample size and the fact that solely women were examined. Further studies with larger sample sizes and different sex are needed to confirm these findings. The investigations prove that growth factor release is detectable in both liquid and solid PRF matrices in comparable concentrations to previous studies.

## Data Availability

The data presented in this study are available on request from the corresponding author. The data are not publicly available due to privacy.
